# Targeting Nrf2 for the treatment of Duchenne Muscular Dystrophy

**DOI:** 10.1016/j.redox.2020.101803

**Published:** 2020-11-18

**Authors:** Stephanie Kourakis, Cara A. Timpani, Judy B. de Haan, Nuri Gueven, Dirk Fischer, Emma Rybalka

**Affiliations:** a College of Health and Biomedicine, Victoria University, Melbourne, Victoria, Australia; b Institute for Health and Sport, Victoria University, Melbourne, Victoria, Australia; c Australian Institute for Musculoskeletal Science, Victoria University, St Albans, Victoria, Australia; d Oxidative Stress Laboratory, Basic Science Domain, Baker Heart and Diabetes Institute, Melbourne, Victoria, Australia; e Department of Physiology, Anatomy and Microbiology, La Trobe University, Melbourne, Australia; f School of Pharmacy and Pharmacology, University of Tasmania, Hobart, Tasmania, Australia; g Division of Developmental- and Neuropediatrics, University Children's Hospital Basel (UKBB), University of Basel, Basel, Switzerland

**Keywords:** Nrf2, Reactive oxygen species, Oxidative stress, Skeletal muscle, Hormesis, Duchenne muscular dystrophy

## Abstract

Imbalances in redox homeostasis can result in oxidative stress, which is implicated in various pathological conditions including the fatal neuromuscular disease Duchenne Muscular Dystrophy (DMD). DMD is a complicated disease, with many druggable targets at the cellular and molecular level including calcium-mediated muscle degeneration; mitochondrial dysfunction; oxidative stress; inflammation; insufficient muscle regeneration and dysregulated protein and organelle maintenance. Previous investigative therapeutics tended to isolate and focus on just one of these targets and, consequently, therapeutic activity has been limited. Nuclear erythroid 2-related factor 2 (Nrf2) is a transcription factor that upregulates many cytoprotective gene products in response to oxidants and other toxic stressors. Unlike other strategies, targeted Nrf2 activation has the potential to simultaneously modulate separate pathological features of DMD to amplify therapeutic benefits. Here, we review the literature providing theoretical context for targeting Nrf2 as a disease modifying treatment against DMD.

## Highlights

• Fatal and rare Duchenne Muscular Dystrophy has high unmet clinical need.
• Many downstream effectors of Nrf2 activation are implicated in dystropathology.
• Targeting Nrf2 may represent a novel therapeutic treatment avenue.
• Approved Nrf2 activator drugs could be rapidly integrated into patient care.

## Abbreviations

ROS: Reactive oxygen species
O_2_: Oxygen
ATP: Adenosine Triphosphate
DMD: Duchenne Muscular Dystrophy
Ca^2+^: Calcium
Nrf2: Nuclear factor erythroid 2-related factor 2
MMF: Monomethyl fumarate
DMF: Dimethyl fumarate
DRF: Diroximel fumarate
MS: Multiple Sclerosis
RNS: Reactive nitrogen species
NOX: Nicotinamide adenine dinucleotide phosphate (NADPH) oxidases
XO: Xanthine Oxidase
NOS: Nitric oxide synthase
ETC: Electron transport chain
PGC-1α: Peroxisome proliferator-activated-receptor-gamma-coactivator-1*α*
SOD: Superoxide dismutase
CAT: Catalase
GPx: Glutathione peroxidase
PRDX: Peroxiredoxins
GSH: Glutathione
HO-1: Heme oxygenase-1
NQO1: Nicotinamide adenine dinucleotide phosphate quinone oxidoreductase 1
O_2_^•^^−^: Superoxide anion
*Dys*: Dystrophin
DAPC: Dystrophin-associated protein complex
4-HNE: 4-hydroxynonenal
ARE: Antioxidant response element
KEAP1: Kelch-like ECH-associated protein 1
Cul3: Cullin-3 protein
DJ-1: Protein deglycase (PARK7)
KO: Knockout
SR: Sarcoplasmic reticulum
SERCA: Sarco/endoplasmic reticulum Ca^2+^-ATPase
NF-κβ: Nuclear factor κ-light-chain-enhancer of activated B cells
CO: Carbon monoxide
Fe^++^: Iron
NLRP3: Nod-like receptor protein 3
iNOS: Inducible nitric oxide synthase
PINK1: PTEN-induced kinase 1
TFAM: Mitochondrial transcription factor A
NRF1: Nuclear respiratory factor 1
PQC: Protein quality control
UPS: Ubiquitin proteasome systems
HSP: Heat shock protein
*dko*: Double knockout
CK: Creatine kinase
LC3-II: Light chain 3-II
SC: Satellite cell
STAT3: Signal transducer and activator of transcription 3
Fam3a: Metabolism Regulating Signalling Molecule A
TRPA1: Transient receptor potential, subfamily A1
ASA: Adenylosuccinic acid
PNC: Purine nucleotide cycle
IMP: Inosine monophosphate
SFN: Sulforaphane

## 1 Introduction

Reactive oxygen species (ROS) form under normal physiological conditions due to the partial reduction of molecular oxygen (O_2_) [1] and, despite being harmful by nature, they are fundamentally important for many physiological processes as functional signalling entities [2]. Moderate levels of ROS are continuously produced by skeletal muscles due to their contractile activity and high O_2_ consumption by the mitochondria. Muscle ROS production is amplified during times of metabolic stress when adenosine triphosphate (ATP) demand is high, such as during exercise, and are buffered by multiple antioxidant systems to maintain redox homeostasis [3]. Redox imbalance, when ROS production exceeds the physiological buffering capacity, results in oxidative stress [4], which is implicated in many pathological conditions including the fatal skeletal muscle wasting disease, Duchenne Muscular Dystrophy (DMD).

DMD is a rare, progressive neuromuscular disorder arising from frame-shift mutations in the dystrophin gene [5]. The mutations, and consequential missing protein, compromise stability and integrity of the skeletal muscle membrane causing the progression of muscle damage, inflammation, degeneration, and wasting [6]. Death occurs typically within the third decade of life due to cardiorespiratory failure [5,7]. Currently, there is no cure and standard of care corticosteroid therapy only slows the progression of the disease [8], and often imparts severe side effects making it unsuitable for the treatment of some patients [8,9]. Emerging genome editing technologies such as CRISPR-Cas9 [10] and TALEN [10] systems offer a new avenue for a potential cure of DMD. Although technical challenges such as efficacy, delivery and accuracy of the genome editing components remain to be improved, exon skipping in particular has positively progressed under accelerated approval based upon limited dystrophin expression [[11], [12], [13], [14], [15]]. However, a clinical benefit is not yet established and escalating side-effect profiles have been identified through post-approval surveillance. Thus, a high unmet clinical need remains for new effective therapeutics with favourable side effect profiles that can be rapidly translated into clinical utility.

DMD is a pathophysiologically complex disease with many druggable targets including calcium (Ca^2+^) regulation [16], mitochondria [3,[17], [18], [19]], antioxidant defence systems [3,20,21], inflammatory and immune responses [7,[21], [22], [23]], satellite cell cycling-mediated regeneration [[24], [25], [26]] as well as protein maintenance regulators [[27], [28], [29]]. Experimental therapeutics in the past have mostly focused on just one of these targets and as such, the therapeutic benefit of these approaches has been limited. A superior therapeutic strategy would be to simultaneously modulate each of these targets to amplify remedial potential. Upon activation, one pathway with the capacity to do this is the nuclear factor erythroid 2-related factor 2 (Nrf2) signalling pathway, which is both a canonical and non-canonical inducer of the cytoprotective response that protects cells from oxidative and other stressors [30]. Nrf2 affects key molecular responses, which address the numerous molecular mediators of dystropathology. Currently, several pharmaceutical and nutraceutical activators of Nrf2 are at different stages of clinical development for the treatment of diseases associated with oxidative stress. However, the only potent, safe and well-characterised pharmacological activators of Nrf2 currently approved for human use are the fumaric acid esters: monomethylfumarate (MMF), dimethyl fumarate (DMF) and the MMF pro-drug formulation, diroximel fumarate (DRF), for Multiple Sclerosis (MS) indication; oltipraz, for schistosomiasis indication; and ursodiol, for primary biliary cirrhosis indication. This review will explore the literature establishing a theoretical context for targeted Nrf2 therapy to treat DMD with the view toward future confirmatory clinical trials.

## 2 ROS and skeletal muscle: Friend or foe?

Skeletal muscles contribute to essential bodily functions including movement, respiration, metabolism and hormonal regulation [26]. Effective muscle function is both energetically and mechanically demanding making it highly susceptible to damage, which, if extensive and chronic, can limit mobility and contribute to metabolic disease. Thus, successful maintenance of muscle function throughout life represents an imperative and critical challenge to healthcare.

Highly metabolic tissues like skeletal muscle are a constant source of ROS and reactive nitrogen species (RNS) [1]. ROS/RNS can be produced in numerous cellular compartments such as the endoplasmic reticulum, lysosomes, peroxisomes and most commonly, the cytosol and mitochondria [31,32]. The main source of ROS production occurs in the cytosol through the activity of nicotinamide adenine dinucleotide phosphate (NADPH) oxidases (NOX enzymes), xanthine oxidase (XO) and nitric oxide synthase (NOS) [32,33], and by the mitochondrial electron transport chain (ETC) [32,33]. Since oxidative damage to biomolecules can have detrimental effects on homeostasis and cellular physiology, oxidative stress is suggested to play a critical role in primary myopathies as well as other diseases which notably feature skeletal muscle dysfunction and/or wasting, including: mitochondrial diseases [34], age-related disorders [35] and neurodegenerative diseases [[36], [37], [38]]. Paradoxically, skeletal muscle also relies on ROS/RNS as important signalling molecules to internally regulate cellular function relative to environmental demands. Physical activity, as well as caloric restriction, transiently increases mitochondrial ROS production to induce mitohormesis [39,40]. In this process low, temporal concentrations of mitochondrial ROS act as signalling molecules to initiate a cascade of cellular events that ultimately protect against harmful events [40,41]. For example, during exercise, ROS promotes mitochondrial biogenesis via the peroxisome proliferator-activated-receptor-gamma-coactivator-1*α* (PGC-1*α*) signalling pathway [42] to facilitate increased ATP production capacity that is required during exercise. However, chronic exposure to ROS paired with inadequate buffering capacity, damages muscle [1] and impairs its regeneration [43]. The type of response, and whether it is beneficial or detrimental, depends on numerous variables such as the origin and reactivity of ROS, the tenacity of ROS generation and the cellular antioxidant status [1]. Endogenous antioxidant systems have evolved to mitigate many forms of oxidative/nitrosative stress and are themselves regulated by mitohormesis.

### 2.1 Hormesis: Beneficial adaptations by ROS

Hormesis refers to a process where exposure to a low toxic insult (that would otherwise be damaging at higher doses), induces a beneficial, adaptive effect that is integral to the physiological function of cells and organisms [44]. Hormesis is a foundational concept in evolutionary theory where, to avoid harm, organisms have developed intricate molecular mechanisms to survive stress. As an example, an acute bout of heavy exercise generates enough ROS to potentially hinder muscle energy production and contractile function in the short-term [45], but, is essential for increasing the resistance of musculoskeletal and cardiovascular systems to injury and disease in the long-term [46]. Thus, hormesis promotes the beneficial cellular adaptations of exercise, which could otherwise be considered a toxic insult.

The main classes of hormetic stress resistance proteins that are conserved across tissues and species have been identified and include, but are not limited to, antioxidant enzymes [47] and protein chaperones [48]. Upregulation of the endogenous antioxidant system is the fundamental response to ROS-induced stress and is aimed at quenching superfluous ROS before they can cause harm to tissues. Antioxidant enzymes are involved in the catalytic transformation of ROS and their by-products into stable, non-toxic molecules thus representing critical defence mechanisms against oxidative damage [49]. The endogenous antioxidant defence system includes enzymes such as superoxide dismutase (SOD), catalase (CAT), glutathione peroxidase (GPx) and peroxiredoxins (PRDX), and the tripeptide glutathione (GSH). These antioxidants are abundant in oxidative (Type I and IIa/x) muscle fibres [[50], [51], [52]] and their deficiency, i.e. SOD1 [50,51], is indicative of oxidative stress. A secondary endogenous antioxidant defence system also exists in the inducible Phase II enzymes – these are activated by a plethora of stimuli such as xenobiotics and drugs [53], endogenous inducers including ROS, lipid peroxidation products and RNS [54], and chemical compounds including isothiocyanates and phenols [55]. Heme oxygenase-1 (HO-1) is responsible for the synthesis of non-enzymatic antioxidants present in skeletal muscle (biliverdin and bilirubin) and has been suggested to play an important role in the regulatory mechanism of heme catabolism in skeletal muscle tissue [50]. Nicotinamide adenine dinucleotide phosphate quinone oxidoreductase 1 (NQO1) is highly inducible and plays crucial roles in cellular adaptation to stress [51], acts as a direct superoxide anion (O_2_^•^^−^) reductase [52] and is significantly elevated in skeletal muscles in response to aerobic exercise [52]. Balance between production of ROS and antioxidant enzyme expression and activity plays an important role to maintain redox homeostasis, while an imbalance has been proposed to progress and exacerbate several muscle-related pathologies such as DMD.

Rather than flicking a molecular on/off switch, hormesis drives biological plasticity according to a graded dose response (either a J-shaped or an inverted U-shaped curve) and through the integration of multiple molecular signals and pathways. In this regard and consistent with most dose-response curves, there is a “ceiling response” which defines the capacity of hormesis to resist any one molecular stress signal. However, the “ceiling” is highly influenced by the local environment and thus may vary substantially between healthy and disease states resulting in insufficient hormesis, failure of redox homeostasis and accelerated pathogenesis [56]. This is particularly true for chronic diseases like DMD, which are underscored by a permanent harmful challenge (e.g. driven by the lack of dystrophin) and an inappropriate response to injury (e.g. driven by a hyper-inflammatory and pro-fibrotic response), which critically challenges hormesis. Importantly, there is evidence to suggest that hormesis remains amenable to rheostatic manipulation even once the theoretical “ceiling” has been reached, and this is because hormetic pathways integrate many signals from multiple molecular pathways through canonical, non-canonical and complementary positive and negative feed-back loops. These avenues can be exploited in disease states to re-establish hormetic control: for Nrf2, this is exemplified through the successful application of DMF in the treatment of the dysregulated immuno- and inflammatory responses associated with MS. But can hormesis by overdone? There is burgeoning evidence to suggest that persistent unsolicited Nrf2 overactivation, particularly alongside dysregulated autophagy, can progress cardiac disease despite being initially beneficial for resisting myocardial damage [57]. Thus, a molecular “tipping-point” clearly exists, which is dependent upon the pathological environment and the proper function and/or amenability to modulation of the downstream cytoprotective events that are governed by Nrf2 (i.e. autophagy). This is important to consider in the context of drug-targeting Nrf2 against chronic disease given the longevity of the treatment needed.

## 3 Duchenne Muscular Dystrophy: When ROS become foes

Progressive muscle wasting conditions like the fatal DMD are underscored by chronic ROS production, which exceeds hormetic capacity. Under these conditions, muscle damage, inflammation and degradation drives disease progression. Designated a rare disease, DMD affects 1 in 3500–7000 live male births worldwide [58,59] and is a X-linked recessive disorder arising from mutations in the dystrophin (*Dys*) gene [60], the largest gene in the human genome [5]. *Dys* mutations result in the ablation of dystrophin, an integral structural cytoskeletal protein that stabilises skeletal and cardiac muscle membranes [23,61]. In healthy muscles, the amino-terminus of dystrophin binds to F-actin with the carboxyl terminus binding to the dystrophin-associated protein complex (DAPC) at the sarcolemma [5]. In the absence of dystrophin, the DAPC is destabilized rendering muscle fibres susceptible to stretch-induced damage [5] and elevated intracellular Ca^2+^ influx [62] leading to a series of pathological processes that are responsible for skeletal and cardiac muscle damage, chronic inflammation [5] and necrosis [63]. The hyper-inflammatory environment drives the deposition of non-functional lipid and fibrous tissue resulting in progressive muscle weakness [64]. Dystrophic symptomology, such as muscular weakness and exercise intolerance [65], is observed between the ages of 2 and 5 with loss of ambulation occurring by early adolescence [59]. Thereafter, patients are wheelchair-bound and comorbidities such as scoliosis and muscular contractures rapidly progress [6]. Even with medical care, DMD patients ultimately die from cardiac and/or respiratory failure within the third decade of life [59,61]. There is currently no cure for DMD and its symptomology is treated non-specifically with corticosteroids (prednisone or deflazacort) as standard of care, despite them eliciting often serious side effects, which limit clinical utility [66,67].

DMD pathogenesis is multifaceted and only partially understood. We and others have documented mitochondrial dysfunction [19,62,68,69] and oxidative stress [17,19,[70], [71], [72]] as central features of the disease. Specifically, we discovered that a mitochondrial Complex I deficit was implicit in the cascade leading to energy depletion, which could be partially recovered by re-routing metabolic flux through Complex II (and chemically inhibiting Complex I) [62]. Others have reported similar mitochondrial aberrations [20,73,74] culminating in energy production deficits [62,73], which may influence the capacity of the muscle to mitigate damage and initiate repair since these processes are energetically-expensive. Furthermore, dysfunctional dystrophic mitochondria generate toxic amounts of ROS [17,19,62,68,69,75] resulting in protein and lipid damage via elevated 4-hydroxynonenal (4-HNE) [76], peroxynitrite [75], and oxidative [17] and nitrosative stress [75], all of which drive the dystrophic condition. Despite dystrophic muscle – from human patients and the genetically homologous *mdx* mouse model – expressing higher levels of antioxidants including SOD and CAT during peak muscle damage periods [[77], [78], [79]], overall antioxidant capacity appears to be insufficient to mitigate ROS as evidenced by lipid [80] and protein [81] peroxidation and hypersecretion of pro-inflammatory cytokines [22,82]. Antioxidant treatment of dystrophic *mdx* mice showed demonstrable benefits in maintaining contractile function and muscle mass [[83], [84], [85], [86], [87], [88], [89], [90], [91]] whilst reducing oxidative damage and inflammation [[83], [84], [85], [86], [87], [88], [89], [90],^92^,^93^] (as summarised in Table 1). As well as having direct ROS-scavenging activity, most of these compounds have also been shown to activate Nrf2 to some degree [94] suggesting that their therapeutic potential depends upon pro-oxidant activity or other co-factors which drive Nrf2-dependent hormesis.

**Table 1:** Antioxidant treatments with pre-clinical efficacy in the mdx mouse model of DMD.

Class	Compound	Dosage	Summary of effects
Direct Nrf2 Activator	Curcumin	0.1, 0.5 or 1 mg/kg daily for 10 days	Improved sarcolemmal integrity, muscle strength and histopathological features; decreased inflammation with all doses - effects most apparent at 1 mg/kg^87^
	Sulforaphane	2 mg/kg daily for 8 weeks	Increased muscle mass and function and expression of Phase II enzymes [97]
		2 mg/kg daily for 4 weeks	Decreased inflammation and increased HO-1 expression [98]
		2 mg/kg daily for 3 months	Reduced inflammation and skeletal and cardiac muscle fibrosis [99]
Antioxidant (with possible hormetic Nrf2 activation)	Adenylosuccinic acid (ASA)	300 mg/mL in drinking water for 8 weeks	Improved histopathological features and mitochondrial pool viability; reduced mitochondrial ROS [19]
	Idebenone	200 mg/kg via oral gavage for 9 months	Reduced cardiac inflammation and fibrosis, increased cardiac function, prevented dobutamine-induced cardiac mortality, increased voluntary wheel-running [100]
	Melatonin	30 mg/kg daily for 11 days	Improved muscle function and muscle redox status [89]
	N-acetylcysteine	2% in drinking water for 6 weeks	Reduced oxidative stress and inflammation and improved overall muscle function [[84], [85], [86]]
		1% in drinking water for 14 days	Improved contractile muscle function and reduced infiltration of collagen and inflammatory cells [90]
	Resveratrol	4 g/kg in food daily for 32 weeks	Decreased ROS and oxidative damage with increased muscle mass [88]
		100 mg/kg every second day for 8 weeks	Improved muscle function but had no effect on oxidative capacity despite slight improvements in muscle pathology [91]
		0.4 g/kg in food daily for 56 weeks	Ameliorated cardiomyopathy with significant reductions in ROS levels [93]
	Taurine	16 g/kg daily in drinking water for 6 weeks	Decreased inflammation and protein oxidation [92]
	Vitamin C	200 mg/kg daily via oral gavage daily for 14 days	Reduced muscle fibre damage, oxidative stress and inflammation [101]
	Vitamin E	40 mg/kg via oral gavage daily for 14 days	Reduced muscle fibre damage, oxidative stress and inflammation and improved membrane repair [102]

Nrf2 is the primary regulator of the endogenous antioxidant response and several Nrf2 activators, or molecules that specifically activate Nrf2-dependent signalling, have been identified/developed. Direct Nrf2 activation may provide a more efficient alternative to indirect modulation through pro-oxidant production to ensure that the magnitude and durability of the endogenous antioxidant response is enough to sustain hormesis and produce a durable benefit in chronic disease states. This is particularly true for the clinically-established Nrf2 activator drugs (e.g. the fumaric acid esters, oltipraz and ursodiol) since many “natural” compounds with antioxidative activity have poor bioavailability and therefore limited translational efficacy (as has been purported for curcumin [95] and the polyphenols [96]). Hence pharmacological induction of the endogenous antioxidant response of Nrf2 will be the forward focus of this review.

## 4 Targeting Nrf2 and its downstream effectors

**Fig. 1:**
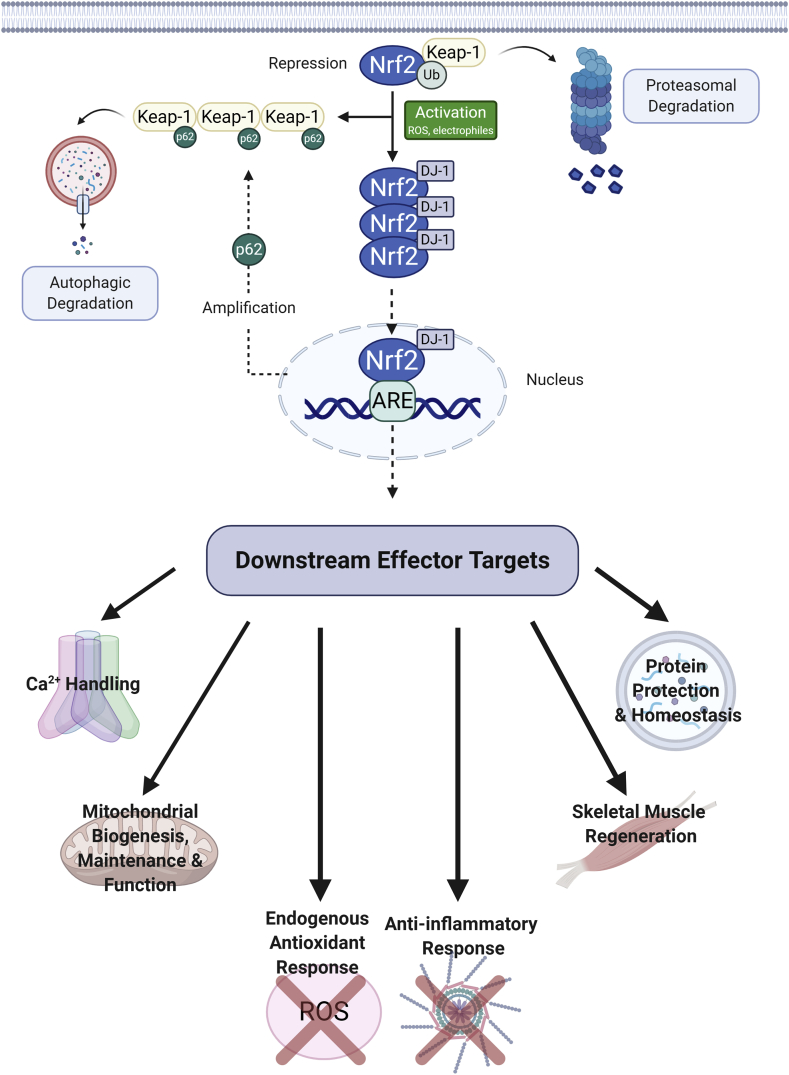
***Nuclear erythroid 2-related factor 2 (Nrf2)/Kelch-like ECH-associated protein 1 (Keap1) signalling pathway and its downstream target effects.*** Under normal conditions, transcription factor Nrf2 is sequestered and degraded in the cytoplasm by its repressor Keap1 and proteosomal degradation, respectively. Activation of Nrf2 in response to stress signals such as reactive oxygen species (ROS) results in partial or complete dissociation of Nrf2 from Keap1. This allows dissociated or newly synthesised Nrf2 and its stabilizer protein DJ-1 to bypass Keap1, which is sequestered by p62, and translocate into the nucleus. Upon nuclear translocation, Nrf2 binds to the antioxidant response element (ARE) modulating the expression of an array of defensive genes including those that encode Phase II detoxifying enzymes such as superoxide dismutase (SOD), nicotinamide adenine dinucleotide phosphate quinone oxidoreductase 1 (NQO1), catalase (CAT) and heme oxygenase-1 (HO-1). This constitutes the endogenous antioxidant response. The activation of Nrf2 leads to broad downstream effects aside from detoxification and cellular defence against potentially harmful ROS, including calcium handling, mitohormesis (including biogenesis, maintenance and function), anti-inflammation (including suppression of pro-inflammatory cytokines), stem cell regeneration (i.e. satellite cell proliferation) and protein protection and homeostasis (namely, autophagy and heat shock protein expression). Nrf2 can amplify its own activation pathway by increasing p62 expression, which targets Keap1 and promotes degradation of the p62-Keap1 aggregate through autophagy. Created with BioRender.com

The Nrf2 signalling pathway has been associated with the regulation of over 600 target genes [103], 200 of which are recognised to encode cytoprotective proteins [104] and endogenous and exogenous stress responses caused by ROS and electrophiles [105]. The core signalling proteins involved in this pathway are the transcription factor Nrf2, which binds to the antioxidant response element (ARE) in the regulatory regions of target genes [106] and Kelch-like ECH-associated protein 1 (Keap1), a Nrf2 repressor, that promotes Nrf2 degradation via the ubiquitin proteasome pathway [106]. Keap1 is rich in cysteine residues that attaches to cytosolic actin and associates with cullin-3 protein (Cul3) to form the Cul3-based E3 ubiquitin ligase complex, which targets substrates for ubiquitin-dependent degradation by the 26S proteasome [107]. Under normal conditions, Nrf2 is bound to two Keap1 molecules [106] and is ubiquitinated by the Cul3-based E3 ubiquitin ligase complex resulting in rapid degradation of constitutively synthesised Nrf2 via the proteasome [105,108]. However, a small amount of Nrf2 avoids this complex allowing for translocation of the transcription factor to the nucleus in order to mediate basal ARE-dependant gene expression [106]. Under stress conditions, canonical Nrf2 inducers, including ROS, electrophiles, toxins and pharmacological agents, alter the cysteines on Keap1, which inhibits Nrf2 degradation in one of two ways. In the first instance, modification of specific Keap1 cysteine residues leads to conformational changes and partial detachment of Nrf2 from Keap1, thus disrupting ubiquitination and degradation of Nrf2^109^. Alternatively, complete disruption of Keap1 and Cul3 interaction in response to electrophiles allow Nrf2 to escape proteasomal degradation [106,109]. In both of these models, Keap1 and its repressor activity, is quenched allowing newly synthesised Nrf2 proteins and stabilizer protein deglycase (DJ-1/PARK7) to translocate into the nucleus where it can bind to the ARE and drive the expression of antioxidant enzymes [105,110] (Fig. 1). Additionally, a number of mechanisms where the Nrf2 pathway is non-canonically activated have also been described: (1) hypermethylation of Keap1, which reduces Keap1 mRNA expression and causes accumulation of Nrf2 in the nucleus [111,112]; (2) loss of fumarate hydratase enzyme activity (catalyses reversible hydration of fumarate to malate [113]), or alternatively, supplemental fumarate, which induces fumarate-dependent succination of Keap1, disrupts Nrf2/Keap1 binding and results in accumulation of Nrf2 [114]; and (3) accumulation of disruptor proteins p21 (directly interacts with motifs on Nrf2 competing for binding with Keap1 [115]) and p62 (directly binds to Keap1 inhibiting Nrf2 interaction and promoting autophagic degradation of Keap1 [115]), which lead to nuclear translocation of Nrf2. Importantly, Nrf2 also induces p62 expression, resulting in the sustained activation of Nrf2 via a positive feedback loop [29]. Nrf2 regulates the transcription of enzymes involved in Phase II detoxification of exogenous and endogenous products (ROS and xenobiotics) and heme metabolism, playing a pivotal role in maintaining redox homeostasis in the cell. As well as these responses, Nrf2 is also involved in other cellular processes including Ca^2+^ regulation, inflammation, intermediary metabolism, autophagy, protein folding responses and stem cell quiescence (as summarised in Fig. 1). The many modes of regulation of Nrf2 activity and the current understanding of Nrf2-mediated transcriptional control will be outlined hereafter.

### 4.1 Targeting Ca^2+^-mediated pathology in DMD through Nrf2

Although the primary deficiency of dystrophin instigates the pathogenesis of DMD, the pathophysiological events that render muscle fibres more susceptible to necrosis are not fully understood. One proposed mechanism implicates abnormal intracellular Ca^2+^ homeostasis owing to disruption of sarcolemmal integrity and increased leak channel activity, which over time leads to progressive fibre degeneration and skeletal muscle weakness [116]. While transient increases in intracellular Ca^2+^ concentrations can induce beneficial muscle adaptations such as increased mitochondrial biogenesis [117] through hormesis, chronically increased intracellular Ca^2+^, which is evident in dystrophic muscle [118,119], can have detrimental effects on muscle function. Ca^2+^ accumulation impairs autophagy [120]; induces mitochondrial Ca^2+^ accumulation [121], permeability transition and apoptosis signalling [122]; and activates the protease, calpain, to degrade skeletal muscle architecture [122]. It has been proposed that Nrf2 can alter intracellular Ca^2+^. Nrf2 knockout (KO) mice present with impaired Ca^2+^ release and uptake kinetics in which the time-to-peak tension is halved. In these animals, the time-to-peak force and half-relaxation time are both doubled compared to Nrf2 positive mice [123]. This suggests that Nrf2 facilitates Ca^2+^ handling as well as sarcoplasmic reticulum (SR) storage relative to the climatic Ca^2+^ concentration. An additional relevant finding involves the GPx8 protein, a GPx that is regulated by Nrf2^124^ and selectively modulates Ca^2+^ storage and fluxes [125]. Nrf2 silencing results in decreased GPx8 and dysregulated intracellular Ca^2+^ leading to cell death, whereas Nrf2 activation via DMF [124] and resveratrol [126] restores Ca^2+^ homeostasis [124] and enhances sarco/endoplasmic reticulum Ca^2+^-ATPase (SERCA) activity [126] and re-uptake to facilitate muscle contraction. Since DMD is partly characterised by chronic Ca^2+^-induced skeletal muscle wasting, Nrf2 activation could effectively rescue functional impairments and convey therapeutic benefits for DMD patients.

### 4.2 Targeting inflammation in DMD through Nrf2

The redox-sensitive signalling Nrf2/Keap1 pathway (Fig. 1) plays a key role in the maintenance of cellular homeostasis under stress and inflammatory conditions. Nrf2 activation can attenuate inflammation via modulation of nuclear factor κ-light-chain-enhancer of activated B cells (NF-κB) [28,98,[127], [128], [129]], a family of transcription factors involved in inflammation, immune responsiveness and apoptosis [129]. NF-κB has been linked to hyperinflammatory diseases and is elevated in the skeletal muscles of DMD patients from infancy, driving inflammation and muscle degeneration [98,130]. Before clinical symptoms occur in DMD, strong activation of components of the innate immune system, including altered signalling via NF-κB, has been reported [82]. Increasing evidence links membrane instability with the release of cytoplasmic contents into the extracellular space [82,131], while the segmental degeneration and regeneration of myofibers [7,131] sustain this chronic activation of the innate immune system. In addition to individually affecting several signalling pathways for maintaining redox homeostasis, Nrf2 and NF-κB cross-talk to further amplify the levels of important redox modulators under basal and disease conditions [132].

Nrf2 also exerts its anti-inflammatory effect via the activation of HO-1 and its metabolism of heme [133,134]. HO-1 is at the core of Nrf2-mediated NF-κB inhibition and catalyses the degradation of heme into carbon monoxide (CO), free iron (Fe^++^) and biliverdin which is then converted to the antioxidant, bilirubin, by biliverdin reductase [30,130]. The role of HO-1 has not been extensively addressed in DMD however the ablation of HO-1 in the *mdx* mouse severely aggravates muscle damage and inflammation [135] whereas pharmacological induction of HO-1 inhibits NF-κB and protects against muscle damage [136]. Nrf2 has been shown to bind in the vicinity of the IL-β and IL-18 genes to decrease their transcription [137], thereby suggesting a direct inhibitory effect on nod-like receptor protein 3 (NLRP3) inflammasome priming. *Mdx* mice exposed to low doses of CO demonstrate reduced muscle damage and atrophy [136] based on HO-1 induction. Nrf2 activation could possibly have a greater influence on the dystrophic phenotype as it not only induces HO-1, but also several other antioxidant enzymes. These data highlight that pharmacologically inducing Nrf2 can alleviate muscle inflammation in *mdx* mice via the inhibition of the NF-κB signalling pathway. Increased HO-1 activity also inhibits NF-κB-mediated transcription of pro-inflammatory cytokines and chemokines [137], matrix metalloproteinases [138], cell adhesion molecules [139,140], and inflammatory enzymes such as cyclooxygenase-2 [141,142] and inducible NOS (iNOS) [141,142]; most of which are overexpressed in dystrophic muscles [[143], [144], [145], [146], [147]]. Inflammation is a key component of the initiation and progression of diseases such as DMD, thus, activating the integrated signalling network involving Nrf2, NF-κB and HO-1 is a logical approach in DMD treatment.

### 4.3 Targeting mitochondrial dysfunction in DMD through Nrf2

Together with its cytoprotective activity against oxidants and other toxic stressors, increasing evidence supports the critical role of Nrf2 to maintain mitochondrial function [148]. Nrf2 is suggested to influence mitochondrial function via the modulation of substrate utilisation during respiration [149]. Nrf2 KO mice present with elevated ROS production, mitochondrial depolarisation, decreased ATP levels and impaired respiration and fatty acid oxidation [[149], [150], [151]], all of which can be rectified following Nrf2 activation [149] or KO of Keap1 [150]. Moreover, Hayashi et al. demonstrated that the canonical Nrf2 activator, DMF, increases mitochondrial DNA and mitochondrial complex mRNA expression in human fibroblasts and wild-type mice [152]. In the same study, DMF was also shown to rescue mitochondrial respiratory chain deficiency in fibroblasts of MS patients [152].

Mitochondrial integrity is also pivotal for mitochondrial function and cell survival. Mitophagy selectively removes excess or damaged mitochondria, maintaining integrity of the pool [93] via the PTEN-induced kinase 1 (PINK1)/Parkinson juvenile disease protein 2 (Parkin) pathway. PINK1 accumulates on the outer membrane of the damaged mitochondria activating Parkin which functions as an E3 ubiquitin ligase [153,154]. Parkin is recruited to the dysfunctional mitochondria, ubiquitinating outer mitochondrial membrane proteins to trigger selective autophagy via the multi-functional protein, p62 [93,153,154]. p62 expression is regulated in a Nrf2-dependant manner, forming a positive feedback loop to amplify its Nrf2-activator function [93]. In *mdx* mice, mitophagy is impaired due to defective PINK1/Parkin signalling [153,155]. Furthermore, Murata et al. demonstrated that stress-damaged mitochondria induce Nrf2-dependant transcription of the PINK1 gene [156], indicating that PINK1 expression is positively regulated by Nrf2. In regard to mitochondrial function, p62 plays a protective role by increasing the translocation of mitochondrial transcription factor A (TFAM) [157], which is vital to mitochondrial DNA replication and transcription. In turn, deficiency of p62 exacerbates defects in mitochondrial membrane potential and energetics ultimately leading to mitochondrial dysfunction [157]. This is demonstrated by p62 KO mice that exhibit a rapid decline of mitochondrial function and present with an accelerated aging phenotype accompanied by severe oxidative stress [158]. It has also been proposed that coordinated induction of HO-1 [159] and NQO1 [158] via activation of Nrf2 induces nuclear respiratory factor 1 (NRF1) expression to elicit the biogenesis of healthy mitochondria. Thus, Nrf2 affects crucial aspects of mitochondrial physiology as evidenced by the multitude of pathological and aging-related disorders associated with diminishing mitochondrial dysfunction and concomitant reduction in/suppression of Nrf2 signalling [148,151,152,158]. Since we have established mitochondrial dysfunction as a driver of DMD [18,62], targeting this aspect through Nrf2 could be therapeutically beneficial for attenuating the disease.

### 4.4 Targeting proteostasis in DMD through Nrf2

Complementing the regulation of antioxidant responsiveness, Nrf2 also prepares the cell for toxic insult by amplifying protein quality control (PQC) measures. ROS, particularly, cause damage to proteins and prevent their proper assembly/reassembly and function, resulting in aggregation and protein conformational disease [160]. To combat this and maintain integrity of the proteome, Nrf2 induces molecular chaperones, the first line defence in proteostasis, to unfold misfolded proteins. But for terminally misfolded and aggregated proteins, degradation and disposal are the only solutions – this occurs through either the calpain and ubiquitin proteasome systems (UPS) or through autophagy, respectively. In this manner, Nrf2 mitigates oxidative injury if ROS quenching cannot be achieved through endogenous antioxidant system-mediated hormesis [161].

Heat shock proteins (HSPs) are highly conserved molecular chaperone proteins that play a role in the assembly, aggregation, transport and folding of proteins [162]. They are induced by stressors including heat, oxidative stress, hypoxia and UV irradiation [163] and upregulated by Nrf2 [164,165]. As well as chaperoning, it is suggested that several HSPs expressed in skeletal muscle play an essential role in muscle development and regeneration [166,167]. The most extensively studied HSP in skeletal muscle is HSP72, which protects and preserves SERCA expression and inhibits inflammatory mediators [163]. Pharmacological (small molecule) HSP72 induction has demonstrated efficacy against the gold standard *mdx* mouse model of DMD, as well as against the more severe *dys*^*-*^*/utr*^*-*^ double *KO* mouse [168]. HSP72 induction reduced creatine kinase (CK) levels – a clinical marker of muscle damage and disease progression – and improved the strength and function of *mdx* muscles [168], while in *dys*^*-*^*/utr*^*-*^ mice, it reduced kyphosis, improved dystrophic muscle pathophysiology and extended lifespan [168]. The capacity for HSP inducers to modify disease pathology highlights the strengths of this therapeutic avenue. We postulate that teamed with the other potentially disease modifying effects elicited by Nrf2, the therapeutic benefit of HSP induction through Nrf2 activation could be amplified further.

Akin to molecular chaperoning, Nrf2 activation governs autophagy-mediated protein and organelle maintenance and proteasomal capacity [169]. In mouse models, autophagy deficiency induces metabolic impairment and accumulation of abnormal organelles, lipids and autophagy-specific substrates such as p62 [170]. Excessive p62 protein accumulation is cytotoxic, escalating both inflammation and oxidative stress [171]. A non-canonical mechanism of Nrf2 activation induced by autophagy deregulation has recently been described [171], differing from the previously described canonical pathway [105]. Accumulated p62 binds to Keap1 hindering its inhibitory effect on Nrf2 [171], which results in the activation of Nrf2 target genes. Autophagy maintains the integrity of the Nrf2/Keap1 pathway by governing Keap1 turnover. In DMD, autophagy is severely impaired in both human patients [172,173] and *mdx* mice [[172], [173], [174]] shown by reduced expression of light chain 3-II (LC3-II) [70,173] and p62 aggregation [29], indicative of the accumulation of damaged protein aggregates owing to reduced autophagic flux [173]. Importantly, the PQC system in *mdx* muscles remains amenable to therapeutic intervention [175]. However, whether autophagy can be targeted through Nrf2 activation as a therapeutic intervention for DMD remains to be investigated.

The proteasome is implicated in the removal of normal and abnormal, denatured or otherwise damaged proteins [169]. Adaptation to oxidative stress involves increased expression of the 20S proteasome [176], and, both insufficient proteolytic capacity or increased oxidant production can result in compromised cellular function [169]. Supressing Nrf2 induction significantly limits the adaptive increases in cellular proteolytic capacity including the 20S proteasome [169]. In contrast, Nrf2 inducers largely increase 20S proteasome expression and cellular resistance to oxidative stress [169], suggesting adaptation is dependent on Nrf2 upregulation and its nuclear translocation. The UPS system is notoriously upregulated in dystrophin-deficient muscle and suppression of proteasomal activity has been pursued to mitigate muscle wasting [177]. This strategy is clearly in conjecture with Nrf2-mediated proteasomal amplification. However, despite successful treatment of *in vivo* animal [[177], [178], [179]] and *ex vivo* human models [177] of DMD with UPS inhibitor drugs, long term human treatment is associated with notable side effects and toxicity [180]. These conflicting data highlight the importance of an active proteasomal system in muscle maintenance, particularly in disease states, as well as the complexity of the pathways that regulate it. Recent data has shed light on this story. NF-κB driven stress signalling appears to orchestrate re-routing of mis-/un-folded protein aggregates into autophagy in favour of the 26S proteasome [181]. Re-establishing normal proteasomal/autophagic balance, rather than inhibiting the UPS, thus might prove a better therapeutic strategy against DMD and could be achieved through Nrf2 activation.

### 4.5 Targeting tissue repair and regeneration in DMD through Nrf2

In addition to cellular protection, Nrf2 is an important regulator of tissue repair and regeneration [182]. Skeletal muscles harbour a population of “satellite” stem cells (SCs) which through induction of the myogenic program factors, MyoD and myogenin [25,183], undergo proliferation, differentiation, fusion and maturation to facilitate muscle repair. Nrf2 plays an important role in muscle regeneration as it prolongs SC proliferation via the upregulation of MyoD, and suppresses SC differentiation by downregulating myogenin [184]. Nrf2 ablation reduces MyoD mRNA and protein expression, delaying muscle regeneration following injury [185]. Signal transducer and activator of transcription 3 (STAT3) regulates SC fate choices during regeneration by promoting SC progression into committed myogenic progenitors and drives differentiation [186]. In conjunction with STAT3, Metabolism Regulating Signalling Molecule A (Fam3a) acts downstream to regulate SC myogenic lineage progression and development of skeletal muscle *in vivo* [183]. Nrf2 stimulates SC proliferation and differentiation by inducing STAT3-mediated secretion of Fam3a to switch the metabolic phenotype of SCs in favour of mitochondrial oxidative phosphorylation and their commitment to myogenic lineage [183]. In dystrophic muscle, loss of STAT3 leads to dysregulation of numerous genes, depletion of SCs as well as heightened inflammation and fibrosis [187]. Transient receptor potential, subfamily A1 (TRPA1) channel activation has also been shown to enhance vital aspects of skeletal muscle repair including promotion of myoblast migration, fusion and potentially SC activation [188] through Ca^2+^-dependent events. Potent Nrf2 activator, DMF, has been shown to directly activate TRPA1 in immune cell populations [189], however it is unknown whether this effect extends to skeletal muscle. Indeed, studies have revealed that injection of freshly isolated SC subsets into dystrophin deficient *mdx* mice renew the endogenous stem cell pool and contribute to improving efficiency of subsequent injury repair [[190], [191], [192]]. This demonstrates that therapies which enhance muscle stem cell activity provide an attractive therapeutic avenue for DMD – pharmacological therapeutics perhaps even more so since muscle stem cell transplant therapy is difficult to administer to the entire skeletal muscle mass for systemic benefit.

## 5 The case for therapeutic pharmacological Nrf2 activation against skeletal muscle wasting

Nrf2 as an exploitable drug target to treat chronic disease is an emerging area of research [152,[193], [194], [195], [196], [197], [198], [199]] since it is capable of inducing an array of downstream target effectors relevant to the pathogenesis of many chronic disease states. There are numerous pharmaceutical and nutraceutical activators of Nrf2 with only a few approved as disease modifying treatment of MS: DMF [200,201], MMF and DRF; schistosomiasis: oltipraz; and primary biliary cirrhosis: ursodiol. Several other molecules are in various stages of development for the treatment of progressive degenerative diseases with neurological and skeletal muscular symptomology (summarised in Table 2) including Alzheimer's Disease, Amyotrophic Lateral Sclerosis, Motor Neuron Disease, Rheumatoid Arthritis and Diabetic Nephropathy.

**Table 2:** Summary of Nrf2 activators that are either approved for human use or have been, or are currently being, examined via human/animal trials.

	Compound	Disease	Phase of Trial	Trial Identifier
Approved for Human Use	Dimethyl Fumarate	Multiple Sclerosis	Approved	N/A
		Psoriasis	Approved	
	Diroximel Fumarate	Multiple Sclerosis	Approved	
	Monomethyl Fumarate	Multiple Sclerosis	Approved	
	Oltipraz	Schistosomiasis	Approved	
	Ursodiol	Primary Biliary Cirrhosis	Approved	
Current Human Trials	Bardoxolone Methyl	Chronic Kidney Disease/Type II Diabetes	Phase III [202]	NCT01351675
	Curcumin	Alzheimer's Disease	Phase II	NCT00099710
		Asthma	Phase II	NCT04353310
	Diacetylbis(N(4)-methylthiosemicarbazonato Copper (II) (CuATSM)	Amyotrophic Lateral Sclerosis/Motor Neuron Disease	Phase III	NCT04082832
		Rectal Cancer	Phase II	NCT03951337
	Dimethyl Fumarate	Cutaneous T Cell Lymphoma	Phase II	NCT02546440
		Glioblastoma Multiforme	Phase I	NCT02337426
		Rheumatoid Arthritis	Phase II	NCT00810836
	Omaveloxolone (RTA 408)	Friedreich's Ataxia	Phase II	NCT02255435
		Melanoma	Phase II	NCT02259231
		Mitochondrial Myopathy	Phase II	NCT02255422
	Resveratrol	Alzheimer's Disease	Phase II [203]	NCT01504854
		Endometriosis	Phase IV [204]	NCT02475564
		Huntington Disease	Phase III	NCT02336633
		Type II Diabetes	Phase I	NCT01677611
	Sulforaphane	Autism Spectrum Disorder	Phase III	NCT02654743
		Chronic Obstructive Pulmonary Disease	Phase II [205]	NCT01335971
		Schizophrenia	Phase II	NCT02810964
Animal Trials	Monomethyl Fumarate	Sickle Retinopathy [206]		
	Resveratrol	Progressive Renal Injury [207]		
	Sulforaphane	Duchenne Muscular Dystrophy [[97], [98], [99]]		
		Breast Cancer [208]		
		Prostate Cancer [209]		

While there are currently no clinical trials investigating Nrf2 activators against skeletal muscle wasting, or specifically, for therapeutic intervention in neuromuscular disease, there is a growing data set which demonstrates that targeting Nrf2 could be beneficial. One of the most debilitating age-associated alterations is the progressive loss of fat-free skeletal muscle mass, a phenomenon commonly known as sarcopenia [210]. Eventual loss of muscle mass and strength, in conjunction with increased adiposity, may occur because of metabolic changes related to a natural age-related decline in physical and biological properties as well as a sedentary lifestyle. Sedentariness can reduce resistance to stressors such as ROS [210], and is a feature of progressed DMD when the magnitude of muscle mass and functional loss leaves sufferers disabled [6], thus perpetuating sedentary atrophy. Overexpression of antioxidant genes such as CAT in model organisms were able to modify oxidative stress tolerance and extend lifespan by up to 50% [211,212]. Therefore, signalling pathways such as the Nrf2-ARE pathway that regulate the antioxidant response could be plausible modifiers of the aging process.

Distinct similarities between Nrf2 KO mice and aged mice are evident. Both exhibit intense sensitivity to various acute and chronic stressors [213], suggesting that disruptions in Nrf2/Keap1 redox signalling may occur throughout the aging process. Safdar et al. investigated the role of the Nrf2/Keap1 signalling pathway in aged skeletal muscle (50–75 years old) of healthy individuals and the modulatory capacity of an active lifestyle on the Nrf2 response [214]. Active elderly subjects showed increased total Nrf2 content, decreased Keap1 protein and activation of Phase II enzymes indicating that the Nrf2-ARE pathway is still capable of hormetic adaptations in response to increased oxidative stress. In contrast, sedentary elderly subjects exhibited decreased Nrf2 function and increased lipid peroxidation suggesting dysfunctional Nrf2/Keap1 signalling. Similar findings are observed in aged Nrf2 KO mice (>24 months) with increased ROS production, ubiquitination, apoptosis and oxidative damage evident in skeletal muscle [196]. Another study concluded that nuclear import of Nrf2 is impaired in older adults (>63 years) compared to young adults and that gene expression of downstream antioxidant targets and Nrf2 is repressed [215]. These studies highlight the importance of the Nrf2/Keap1 signalling pathway in maintaining the cellular redox state in elderly/aged skeletal muscle, but perhaps most profoundly, that pharmacological Nrf2 activation should be considered in sub-populations of individuals who cannot voluntarily exercise due to disease and disability to protect skeletal muscle mass.

Akin to sarcopenia, Nrf2 expression in DMD patients declines as the disease progresses [70] suggesting that the endogenous Nrf2-mediated cytoprotective response declines too. In infants (0–2 year old) and young (2–9 year old) DMD boys, Nrf2 protein expression and activation declines with age/weight-bearing activity/disease progression, as does downstream Phase II enzyme expression [70]. In the skeletal muscle of infant DMD patients in particular, gene expression of Nrf2 and Phase II enzyme expression (i.e. NQO1 and SOD) is notably higher than in healthy age-matched controls but paradoxically, HO-1 is downregulated [70]. These data suggest oxidative stress in skeletal muscle early in the disease but subnormal Nrf2-dependent anti-inflammation signalling through HO-1. This study defines the critical role of oxidative stress on muscle wasting, particularly concerning the early pathogenic mechanisms underlying DMD. It also begs the question whether Nrf2 can induce hormetic adaptations in the dystrophic environment necessary to elicit a therapeutic response through pharmacological activation?

To answer this, our group recently investigated the therapeutic benefits of Nrf2 activation in the *mdx* mouse following 8-week treatment with the fumarate-generating metabolite, adenylosuccinic acid (ASA) [19]. ASA is a product of the Purine Nucleotide Cycle (PNC), which is activated during times of stress to drive the recovery of ATP from inosine monophosphate (IMP) [216]. The enzymatic cleavage of ASA also yields fumarate that is transported into the mitochondria (via the malate-aspartate shuttle), expanding the tricarboxylic acid cycle and specifically driving the malate > fumarate > succinate flux through Complex II to augment ATP production [216]. Treatment with ASA improved mitochondrial viability, reduced lipid and connective tissue infiltration, and significantly improved histopathological features of murine DMD. Originally, it was thought that ASA exerted it effects metabolically but we have since identified that ASA upregulates Nrf2 protein expression (Rybalka et al., *Manuscript in review* [217]). In immortalised human DMD cells, ASA has antioxidant effects through reduction of mitochondrial superoxide [19]. Our data is consistent with Sun et al. who treated dystrophic mice with the canonical Nrf2 inducer, sulforaphane (SFN) [[97], [98], [99]], which mediates its effects through induction of NQO1 [218] and HO-1 [193]. These studies demonstrated that SFN treatment of the *mdx* mouse induced NQO1 and HO-1 expression, increased skeletal muscle mass and force production, decreased NF-κB [98] and the inflammatory cytokine TNF-α [99], and improved sarcolemmal integrity as evidenced by a decrease in Evans Blue Dye permeation [97]. SFN also reduced fibrosis through inhibition of TGFβ/Smad signalling [99]. Thus, Nrf2 activators appear useful to ablate the central mechanistic hallmarks of dystrophic progression.

As yet, there are no trials in DMD patients investigating established Nrf2 activator drugs against the disease course. However, in a 2019 case study, treatment with ASEA Redox Supplement®, a deionised saline water with Nrf2 activator capacity, was shown to attenuate serum biomarkers of DMD progression (CK, CK-MB (cardiac muscle-specific) and magnesium levels) in one young DMD patient. Treatment occurred over 16 months with escalating dosage (4–7 mL/kg/day [219]) and importantly demonstrated no liver toxicity, a side-effect of perpetual genetic Nrf2 induction. While the data generated from the case study are promising, it is important to highlight that the Nrf2 activator supplement was administered in combination with _l_-carnitine, omega-3 fatty acids and a vitamin cocktail which could significantly confound interpretation of the data.

## 6 Balancing the argument: Could too much Nrf2 be detrimental?

Whilst Nrf2 is undoubtedly a strong molecular cytoprotectant, it is important to consider several factors when proposing its chronic induction to treat disease: (1) repressor molecules like Keap1 have been evolutionary conserved to maintain low Nrf2 levels during the basal redox steady state; (2) chronic unsolicited Nrf2 overactivation may tip redox imbalance in the opposite direction by driving reductive stress and pathogenesis; and (3) other non-transcriptional activities relating to Nrf2's sequestration of Keap1 are important for tissue growth, repair and maintenance (e.g. angiogenesis).

Genetic overexpression/overactivation models have provided insight regarding the negative effects of unsolicited Nrf2 activation. In Drosophila, high Nrf2 levels cause developmental lethality, or after inducible activation, altered mitochondrial bioenergetics, diabetes onset and accelerated aging through modulating insulin/IGF signalling to induce a stress tolerance phenotype over a growth and longevity phenotype [220]. In mice, persistent Nrf2 overactivation reduces lifespan through driving stem cell exhaustion [221], causes hepatomegaly, glycogenosis and steatosis [222], and drives cardiomyopathy and heart failure when autophagy is impaired [223]. While these data provide important proof of concept evidence that Nrf2 overactivation can be lethal, it is important to highlight that genetic overexpression/activation and pharmacological activation represent two very different therapeutic approaches. Where pharmacological activators work through supporting hormesis proportionate to the dose administered, the magnitude and persistence of genetic activation overrides hormesis and the adaptive response to environmental stress cues. Indeed, mild genetic Nrf2 overactivation of Drosophila induced cytoprotection, enhanced stress tolerance and extended lifespan, suggesting that functional hormesis is required to elicit beneficial effects and can be achieved through modifying the degree of Nrf2 activation.

Without doubt, the most controversial outcome of prolonged and gain-of-function Nrf2 activation concerns cancer progression [[224], [225], [226]]. It is well established that cytoprotection elicited through Nrf2 activation is important for the suppression of carcinogenesis in the first instance [[227], [228], [229], [230]]. However, Nrf2 activity in aggressively malignant cells promotes tumorigenesis and metastasis [228,[231], [232], [233]], increases resistance to platinum-based chemotherapy [224,[234], [235], [236], [237]] and has been associated with overall poorer prognosis [238]. These studies suggest a protective role of Nrf2 in the early stages of cancer compared to a more advanced stage where Nrf2 overexpression assists cancer cells to adapt to tumorigenic demands. Although there is considerable evidence suggesting that Nrf2 activation is both safe to use and effective against several other diseases, it is essential to further investigate whether chronic, long-term Nrf2 activation therapy increases the risk of cancer development.

Nrf2 activation may also be detrimental by causing reductive stress. There is surmounting evidence that reductive stress, as much as oxidative stress, can promote pathogenesis through abolishing functional ROS signalling and/or increasing reducing equivalents [239,240]. Oxidative modification of cysteines, for example, is essential for controlling protein synthesis and quality control in the endoplasmic reticulum and for the activation of growth factor-mediated protein kinase signalling. Similarly, ROS are essential for the responsive metabolic reprogramming and angiogenesis evoked by hypoxia through HIF1α. Paradoxically, reductive stress appears to eventually propagate oxidative stress through the consumption of reducing equivalents by mitochondria and other ROS producing enzymes in a mechanism that is not entirely clear [239]. This may explain how ROS scavenging antioxidants administered at certain (generally high) concentrations can activate Nrf2 through pro-oxidant activity. In the context of drug targeting for the treatment of DMD, it would be necessary to balance the anti-oxidant/pro-reducing equivalent/pro-oxidant activity elicited by the chosen Nrf2 activator drug through dosage and regimen refinement to ensure efficacy. The successful use of Nrf2 activator drugs against other diseases (e.g. MS) characterised by chronic and excessive oxidative stress and inflammation highlights that this can be achieved in the clinic [241].

Finally, it has recently emerged that Nrf2 elicits other crucial cellular functions independent of its transcriptional activity, which could be abolished by chronic unsolicited Nrf2 activation. Through biologically anchoring Keap1, Nrf2 drives angiogenesis by endothelial cells, and therefore, maintains tissue oxygenation [242]. This is fundamentally important in DMD, because dystrophin is also expressed in vascular endothelial and smooth muscle cells and without it, angiogenesis and muscle oxygenation is already impaired (reviewed in Ref. [243]). In this regard, it would be important to consider the consequence of Nrf2 activation (i.e. dissociation) on Keap1. For example, p62 has been shown to aggregate with Keap1 which might retain Keap1's pro-angiogenic regulatory function independent of Nrf2 but only at a rate consistent with the rate of autophagy activity [244]. This aspect requires further investigation.

## 7 Conclusion

The Nrf2 signalling pathway governs the antioxidant, metabolic and mitochondrial re-programming response to cellular stress and is the target of numerous established and investigational new drugs to treat chronic disease. Pharmacological Nrf2 activators have the potential to therapeutically exploit key targets involved in the pathogenic regulation of diseases such as DMD, which include Ca^2+^ dysregulation, mitochondrial dysfunction, compromised antioxidant responsivity, inflammation and impaired satellite cell-mediated regenerative activity. There is developing pre-clinical evidence in this regard, using established and novel Nrf2 activator compounds which show capacity to attenuate murine (*mdx*) MD. Since we provide here, only a theoretial context for Nrf2-mediated therapy against DMD, further translational investigations on the therapeutic effects and safety of novel, as well as repurposed, modulators of the Nrf2 signalling pathway should be undertaken in clinical trials. Given the success of long-term pharmacological Nrf2 therapy against MS in particular, as well as an isolated case study using an Nrf2 activator supplement in one DMD patient which demonstrated promising effects on disease biomarkers, Nrf2 could be a useful therapeutic target against not only DMD, but also other neuromuscular, neurodegenerative and metabolic diseases.

## Funding sources

This work was supported by funding from 10.13039/501100001784Victoria University and University Children's Hospital Basel.

## Declaration of Competing interest

The authors declare the following financial interests/personal relationships which may be considered as potential competing interests: Emma Rybalka is a scientific consultant to Santhera Pharmaceuticals. Nuri Gueven is a scientific consultant to Santhera Pharmaceuticals. Dirk Fischer is principle investigator for studies on spinal muscular atrophy sponsored by Hoffman-La Roche Ltd.
